# Supplementary material for: Pulmonary and Gastrointestinal Parasitic Infections in Small Ruminant Autochthonous Breeds from Centre Region of Portugal—A Cross Sectional Study
Source: Animals (Basel). 2024 Apr 21;14(8):1241. doi: 10.3390/ani14081241 (PMC11047328; doi:10.3390/ani14081241)
Supplement: Supplementary file 1 [file animals-14-01241-s001.zip › animals-2904120-supplementary new.pdf]

Table S1. Association between sheep Protostrongylidae infection and background information, including animal signalment and parasitological information (n=208).

Variable	Total	Presence n (%)	Absence n (%; %*)	Chi-square (p)
**District**				
Viseu	15	0	15 (7.2; 100.0)	
Guarda	165	28 (13.5; 17.0)	137 (65.9; 83.0)	<0.001
Coimbra	28	12 (5.8; 42.9)	16 (7.7; 57.1)	
**Pasture sharing**				
Yes	105	13 (6.3; 12.4)	92 (44.2; 87.6)	0.011
No	103	27 (13.0; 26.2)	76 (36.5; 73.8)	
**Production purpose**				
Milk	208	40 (19.2; 19.2)	168 (80.8; 80.8)	-
Meat	0	0	0	
**Sex**				
Female	201	38 (18.3; 18.9)	163 (78.4; 81.1)	0.403*
Male	7	2 (1.0)	5 (2.4)	
**Breed**				
Serra da Estrela	208	40 (19.2; 19.2)	168 (80.8; 80.8)	
**Age (months)**				
0-12	3	1 (0.5; 33.3)	2 (1.0; 66.7)	0.475*
>12	205	39 (18.8; 19.0)	166 (79.8; 81.0)	
**Parasitological testing**				
Yes	15	0	15 (7.2)	0.036*
No	193	40 (19.2; 20.7)	153 (73.6; 79.3)	
**Deworming frequency**				
Twice a year	148	26 (12.5; 17.6)	122 (58.7; 82.4)	0.339
Annual	60	14 (6.7; 23.3)	46 (22.1; 76.7)	
**Dewormer**				
Eprinomectin (Eprecis®)	45	20 (9.6; 44.4)	25 (12.0; 55.6)	
Albendazol (Sinvermin®)	13	1 (0.5; 7.7)	12 (5.8; 92.3)	
Mebendazol + Closantel (Seponver plus®)	60	12 (5.8; 20.9)	48 (23.1; 80.0)	<0.001
Ivermectin + clorsulon (Topimec ®; Ivomec F®)	90	7 (3.4; 7.8)	83 (39.9; 92.2)	

%- Percentage of total sheep; %* - Percentage of the number of sheep per category; *Fisher exact test

Table S2. Association between goats Protostrongylidae infection and background information, including animal signalment and parasitological information (n=203).

Variable	Total	Presence n (%, %*)	Absence n (%)	Chi-square (p)
**District**				
Viseu	188	180 (88.7; 95.7)	8 (3.9; 4.3)	0.506*
Guarda	0	0	0	
Coimbra	15	14 (6.9; 93.3)	1 (0.5; 6.7)	
**Pasture sharing**				
Yes	0	0	0	-
No	203	194 (95.6; 96.6)	9 (4.4; 4.4)	
**Production purpose**				
Milk	30	23 (11.3; 76.7)	7 (3.4; 23.3)	<0.001*
Meat	173	171 (84.2; 98.8)	2 (1.0; 1.2)	
**Sex**				
Female	192	183 (90.1; 95.3)	9 (4.4; 4.7)	0.599*
Male	11	11 (5.4)	0	
**Breed**				
Crossed breed Serrana ecotype Transmontana	188	185 (91.1; 98.4)	3 (1.5; 1.6)	<0.001*
Serrana ecotype Jarmelista	15	9 (4.4; 60.0)	6 (3.0; 40.0)	
**Age (months)**				
0-12	18	18 (8.9; 100.0)	0	0.338*
>12	185	176 (86.7; 95.1)	9 (4.4; 4.9)	
**Parasitological testing**				
Yes	0	0	0	
No	203	194 (95.6; 95.6)	9 (4.4; 4.4)	
**Deworming frequency**				
Twice a year	15	9 (4.4; 60.0)	6 (3.0; 40.0)	<0.001
Annual	188	185 (91.1; 98.4)	3 (1.5; 1.6)	
**Dewormer**				
Eprinomectin (Eprecis®)	15	9 (4.4; 60.0)	6 (3.0; 40.0)	<0.001*
Albendazol (Sinvermin®)	173	171 (84.2; 98.8)	2 (1.0; 1.2)	
Mebendazol + Closantel (Seponver plus®)	15	14 (6.9; 93.3)	1 (0.5; 6.7)	
Ivermectin + clorsulon (Topimec ®; Ivomec F®)	0	0	0	

%- Percentage of total goats; %*- Percentage of the number of goats per category; * Fisher Exact Test
